# Nutritional Quality, Voluntary Intake and Enteric Methane Emissions of Diets Based on Novel Cayman Grass and Its Associations With Two *Leucaena* Shrub Legumes

**DOI:** 10.3389/fvets.2020.579189

**Published:** 2020-10-20

**Authors:** Xiomara Gaviria-Uribe, Diana M. Bolivar, Todd S. Rosenstock, Isabel Cristina Molina-Botero, Ngonidzashe Chirinda, Rolando Barahona, Jacobo Arango

**Affiliations:** ^1^Grupo de Investigación BIOGEM, Departamento de Producción Animal, Facultad de Ciencias Agrarias, Universidad Nacional de Colombia, Medellín, Colombia; ^2^International Center for Tropical Agriculture (CIAT), Cali, Colombia; ^3^World Agroforestry Centre (ICRAF), Kinshasa, Democratic Republic of Congo; ^4^Consortium of International Agricultural Research Centers (CGIAR) Research Program on Climate Change, Agriculture and Food Security, Kinshasa, Democratic Republic of Congo

**Keywords:** climate change mitigation, forage diversification, greenhouse gases, livestock, urochloa hybrid pastures

## Abstract

Methane (CH_4_) emissions from enteric fermentation in cattle are an important source of greenhouse gases, accounting for about 40% of all agricultural emissions. Diet quality plays a fundamental role in determining the magnitude of CH_4_ emissions. Specifically, the inclusion of feeds with high digestibility and nutritional value have been reported to be a viable option for reducing CH_4_ emissions and, simultaneously, increase animal productivity. The present study aimed to evaluate the effect of the nutritional composition and voluntary intake of diets based on tropical forages upon CH_4_ emissions from zebu steers. Five treatments (diets) were evaluated: Cay1: *Urochloa* hybrid cv. Cayman (harvested after 65 days of regrowth: low quality); Cay2: cv. Cayman harvested after 45 days of regrowth; CayLl: cv. Cayman + *Leucaena leucocephala*; CayLd: cv. Cayman + *Leucaena diversifolia*; Hay: *Dichantium aristatum* hay as a comparator of common naturalized pasture. For each diet representing different levels of intensification (naturalized pasture, improved pasture, and silvopastoral systems), CH_4_ emissions were measured using the polytunnel technique with four zebu steers housed in individual chambers. The CH_4_ accumulated was monitored using an infrared multigas analyzer, and the voluntary forage intake of each animal was calculated. Dry matter intake (DMI, % of body weight) ranged between 0.77 and 2.94 among diets offered. Emissions of CH_4_ per kg of DMI were significantly higher (*P* < *0.0001*) in Cay1 (60.4 g), compared to other treatments. Diets that included *Leucaena* forage legumes had generally higher crude protein contents and higher DMI. Cay1 and Hay which had low protein content and digestibility had a higher CH_4_ emission intensity (per unit live weight gain) compared to Cay2, CayLl and CayLd. Our results suggest that grass consumed after a regrowth period of 45 days results in lower CH_4_ emissions intensities compared to those observed following a regrowth period of 65 days. Diets with *Leucaena* inclusion showed advantages in nutrient intake that are reflected in greater live weight gains of cattle. Consequently, the intensity of the emissions generated in the legume-based systems were lower suggesting that they are a good option for achieving the emission reduction goals of sustainable tropical cattle production.

## Introduction

The methane (CH_4_) emissions from the livestock sector accounted for about 97 Mt in 2014, corresponding to ~2.7 Gt of equivalent CO_2_ (1). The primary source of CH_4_ emissions in agriculture is enteric fermentation, the process in which the anaerobic digestion of feed in cattle rumen produces CH_4_ through the activity of methanogenic microorganisms, accounting for 44% of total emissions from this activity (2).

Early estimations of daily CH_4_ emissions in animals ranged from 250–500 L per animal (3). These emissions have not only environmental but also productivity implications since energy losses in the form of CH_4_ vary between 2–15% of total gross energy (GE) ingested (4). Although CH_4_ has a warming potential 21 times > CO_2_, its net contribution may be even higher as a greenhouse gas (GHG). However, CH_4_ has a short atmospheric lifetime (10–12 years compared to other greenhouse gases, *i.e*., 120 years for CO_2_, 114 years for N_2_O), so reducing its emission may have short-term benefits (5) and rapid cooling effect in the atmosphere.

The way the animals are fed and the composition of the feed are crucial drivers of CH_4_ production due to enteric fermentation. Major determinants of the amount of enteric CH_4_ produced by cattle are the type, quality, and quantity of consumed forage (6, 7). The quality of the consumed forage is based on its carbohydrates, fat, protein, and mineral composition. The digestibility of the forage depends on the form of carbohydrates. Specifically, forages with high amounts of free sugars and starch are easier to digest, generally have shorter retention times in the rumen and, consequently, are associated with lower CH_4_ production compared to forages with high amounts of structural carbohydrates such as neutral detergent fiber (NDF) and acid detergent fiber (ADF) (8). Forages with high amounts of structural carbohydrates, which increase with sward age, are considered to have lower quality (9, 10). Several previous studies have reported lower enteric CH_4_ emissions with younger compared to older forage swards and attributed this difference to the higher amounts of free sugars and starch in younger forage (10, 11).

In tropical countries, forages are the most economical and practical feed option for cattle, which alongside with proper management, can be available throughout the year and supply adequate nutrition. However, forage nutritional quality is highly variable, and influenced by many factors such as season, age and fertilization (12). Previous studies have reported that high fiber and lignin contents reduce the level of forage voluntary intake of grazing cattle (13, 14). In the lower tropical regions of Colombia it has been reported that the inclusion of legumes such as *Leucaena* sp. is beneficial for increasing cattle productivity as it increases the supply of highly digestible protein (15), reduces the amount of structural carbohydrates and contributes toward increasing the dry matter intake (DMI) and reducing the intensity of CH_4_ emissions per unit DMI and dry matter digested (DMD) (16, 17). Cayman hybrids have been widely adopted by farmers in the Colombian lower tropics because of their tolerance to waterlogging, high forage production and contents of protein (18). However, GHG emissions associated with Cayman hybrids remain largely unknown.

The transition toward the sustainable intensification of cattle production systems may require the promotion and adoption of improved forages (*i.e.*, inclusion of legumes and genetically improved grasses) (19–21). To contribute to sustainable cattle production these improved forages should be associated with an increase in milk and meat productivity and a reduction in GHG emissions and other harmful activities to the environment (*e.g.*, deforestation, soil degradation, and biodiversity loss). Tropical livestock systems based on diverse, well-managed improved forages (including *Leucaena*) could contribute to reduce the emissions of CH_4_ (17), nitrous oxide [N_2_O, (22)] and increase carbon accumulation in aerial biomass and soils (23).

While the reduction of enteric CH_4_ emissions is technically possible, there are still various challenges (*e.g.*, weak policies, financial incentives, and technical assistance) that should be overcome before these technologies can be implemented at a broader scale (21). Once these hurdles are overcome, some of these forage-based technologies could effectively contribute to produce more milk and meat per unit of area and, concurrently, deliver ecosystem services such as climate change mitigation (20). Reducing the high GHG emissions associated with the cattle sector represents an opportunity for countries to advance toward achieving their Nationally Determined Contributions (NDCs) under the Paris Agreement.

We hypothesized that cattle consuming high quality forages have high voluntary intake and low CH_4_ emissions intensities. We also hypothesized that the inclusion of *Leucaena* contributes to improve the quality of the consumed diet and reduces CH_4_ emissions. The present study aims at generating critical technical information on forage quality, voluntary intake, optimal grazing management, and enteric CH_4_ emissions of a recently bred tropical forage-grass (Cayman hybrid), alone and in combination with two legume tree species (*Leucaena* sp.). These diets were chosen because they allowed us to test our hypothesis using forage options common in the lower tropical regions of Colombia, Cayman hybrid, *Leucaena leucocephala* and *Leucaena diversifolia*. We also included *Dichanthium aristatum* which is commonly used for hay during dry periods.

## Materials and Methods

### Study Site

The experiments were conducted in the regional office for Colombia of the Alliance of Bioversity International and the International Center for Tropical Agriculture (CIAT) located in Palmira, Valle del Cauca, Colombia (3°30'7”N; 76°21'22”W) at 1,050 masl. This site has an annual mean temperature of 27°C and rainfall of 1008 mm.

### Diets and Forage Materials

The forages evaluated in this study were from different experimental plots located at CIAT. The soil of these plots was characterized as Cumulic Haplustoll (Soil Taxonomy, USDA 2014) with a silty clay texture (50 % clay in the upper 20 cm soil layer) and pH = 7.5 (24).

Five treatments (diets) were evaluated in the present study: **Cay1**: *Urochloa* hybrid cv. Cayman (harvested after 65 days of regrowth); **Cay2**: cv. Cayman (harvested at 45 days); **CayLl**: cv. Cayman + *Leucaena leucocephala;* **CayLd**: cv. Cayman + *Leucaena diversifolia* and **Hay**: Hay of *Dichantium aristatum* used for comparison as one of the most common feed resources in the region.

In Cay1 and Cay2, animals received *Urochloa* hybrid cv. Cayman-CIAT BR02/1752 (Cayman) of contrasting chemical composition and nutritional quality due to differences in harvesting time (*i.e*., regrowth age of 65 days and 45 days for Cay1 and Cay2, respectively), to simulate two different grazing regimes. Leaves of *Leucaena leucocephala* CIAT 17263 and *Leucaena diversifolia* ILRI 15551 were hand-collected after 58 days of regrowth simulating cattle browsing. *Dichantium aristatum* was collected at 52 days of regrowth.

### Nutritional Quality of Forage-Based Diets

Forages consumed by the cattle (four zebu steers) were analyzed at CIAT's Forage Quality and Animal Nutrition Laboratory [certified by the FAO-IAG proficiency test of feed constituents 2016 and 2017 (25)], to determine their chemical composition and nutritional value. Plant samples were dried at 55°C for 72 h, following the method 6496 of the International Organization for Standardization (26) to determine the content of dry matter (DM); and crude protein [CP = N concentration × 6.25; Kjeldahl AN 3001 FOSS; AOAC (27): method 984.14]; neutral detergent fiber (NDF) and acid detergent fiber (ADF) by the methodologies proposed by Van Soest et al. (28) adapted to an Ankom Fiber Analyzer AN 3805 (Ankom® Technology Corp. USA); gross calorific value using calorimetry was determined per [ISO 9831, (29)] specifications and *in vitro* DMD by the technique of Tilley and Terry (30). Condensed tannins (CT) were determined only for the treatments containing *Leucaena*, according to the method described by Terrill et al. (31).

### Experimental Design and Animals Management

Four zebu steers with an initial weight of 220 ± 18 kg were used in the evaluation of all treatments, which finalized in July 2017. One diet (out of five) was provided to the four animals for 19 days, of which the first 15 days were for adaptation to the diet, in which the animals grazed the experimental plots *ad libitum*. Following the adaptation period to the diet, the animals were moved to the polytunnel for 3 days for acclimatization to the polytunnel conditions. During these 3 days, the steers were subjected to short periods of total polytunnel enclosure during the day. Measurements of CH_4_ emissions were made in closed polytunnels with the individual animals on day 19 of the experimental period. This methodology was used to assess each diet described in section Diets and Forage Materials.

### Polytunnel Conditions

Measurements of CH_4_ were conducted as described by Lockyer (32) and Murray et al. (33). Two polytunnels (Area: 48 m^2^ and volume: 134 m^3^) were used; each of these structures had an entrance for two animals. On the opposite side, there was a 12” fume extractor set at an extraction rate of 0.9 m^3^ s^−1^ to allow the collection of gas samples. Each polytunnel was sub-divided into two independent chambers, each with a volume of 67 m^3^, making it possible to evaluate four independent animals simultaneously. Gas samples were collected every 60 to 90 min from inside and outside each polytunnel chamber over 24 h, starting at 08:00 am, accounting for 18 different sampling times. At the end of each measurement, the polytunnel was opened to release accumulated gas before beginning new gas measurements.

The environmental conditions inside and outside (environmental) the polytunnel were monitored continuously during the experimental period to ensure that the temperature and humidity inside the tunnel did not generate heat stress in the animals. In addition, a solar screenwas installed to provide shade for the polytunnel and prevent high temperatures inside the polytunnel, which would affect the animals' welfare during the hottest hours of the day.

### Methane Concentration in Gas Samples

Every measurement consisted of the collection of gas samples every 20 s using a portable FTIR gas analyzer Gasmet DX4040 (Gasmet Technologies Oy®, Helsinki, Finland). The equipment was calibrated with ultrapure dinitrogen gas grade 5.0 according to manufacturer instructions.

The amount of CH_4_ was calculated using the ideal gas law (34), from the concentration (ppm) reported by the Gasmet® and the total volume of the polytunnel. Additionally, ambient air was sampled each time the sample was taken from inside the tunnel, in order to correct each gas measurement obtained.

### Dry Matter Intake

Forages of the different diets were offered individually to animals in feeders installed inside the polytunnels. These forages were cut directly from the experimental grazing plots and offered fresh without being chopped (Hay was the only treatment that was offered dry). The harvesting of the forages was done to simulate the intake behavior observed when the animals were grazing. All animals had *ad libitum* access to forages, salt, and water throughout the experimental period. No additional concentrates or supplements were added. The voluntary daily intake of each individual animal, for each of the diets was calculated as the difference between the amount of forage offered and rejected.

### Expected Live Weight Gains

Body weight (BW) gains (g per day) were estimated using The Cornell Net Protein and Carbohydrate System-CNCPS® version 6.0 (35). Input variables for the model included animal management, environmental variables, chemical forage composition, initial animal body weight, and DMI.

### Data Analyses

Data were analyzed using the GLM procedure of the software Statistical Analysis Systems®, version 9.2 (36). The experimental design was a randomized complete block design with the animal considered as the block. The animal weight was used as a covariate, to eliminate errors associated with changes in animal weight over time. Multiple comparisons were evaluated using Tukey's HSD test. Relationships between CH_4_ emitted, and DMD or NDF- ADF intake and DMI and nutrient content were analyzed by type II linear regression using PROC REC procedure of SAS.

## Results

### Nutritional Quality and Digestibility of Forage-Based Diets

The evaluated diets showed differences in the contents of nutrients and digestibility (Table 1). Treatments including *Leucaena* (CayLl and CayLd) had two to three times higher CP compared to grass-alone treatments, whereas the late-harvested Cayman grass Cay1 showed the lowest CP among all diets. Contents of NDF and ADF tended to decrease in the treatments in which the Cayman grass was combined with *Leucaena* forages (CayLl and CayLd). Ash content varied between 118 and 175 g kg DM^−1^ for the evaluated diets, while GE intake was, on average, 16.9 MJ kg DM^−1^ for all diets except for the control diet (Hay, 14.1 MJ kg DM^−1^). The digestibility of the diets ranged from 479–618 g kg DM^−1^, with Cay1 and the Hay being the least digestible treatments. Treatments of the same grass (Cayman) that differed in harvesting times showed dissimilar CP contents and digestibility. Cay2 had 46.5% more CP content and 17.3% more digestibility than Cay1 (Table 1). The contents of CT were 28.8 and 32.6 g kg DM^−1^ for *L. leucocephala* and *L. diversifolia*, respectively.

**Table 1:** The nutritional value of five different diets based on tropical-forages (treatments) evaluated offered to Brahman cattle steers.

	**Cay1**	**Cay2**	CayLl^*^	CayLd^**^	**Hay**
DM	391	213	211	238	632
CP, g kg DM^−1^	44.5	83.3	96.2	128.5	62.3
NDF, g kg DM^−1^	709.8	682.2	638.5	580.9	612.6
ADF, g kg DM^−1^	414.2	349.1	359.2	299.3	388.9
Ash, g kg DM^−1^	118.3	121.4	124.5	175.6	140.3
GE, Mj kg DM^−1^	16.2	17.2	16.7	17.5	14.1
IVDMD, g kg^−1^	511	618	610	606	479

* *Content of condensed tannins of Leucaena leucocephala = 28.8 g/kg DM*;

** *Content of condensed tannins of Leucaena diversifolia = 32.6 g/kg DM*.

*DM, Dry matter; CP, crude protein; NDF, neutral detergent fiber; ADF, acid detergent fiber; GE, gross energy; IVDMD, in vitro dry matter digestibility. Cay1 and Cay2 diets correspond to Urochloa hybrid cv. Cayman-CIAT BR02/1752 (Cayman) collected at different times of regrowth (i.e., 65 and 45 days, respectively). CayLl is a mixture of Cay2 and Leucaena leucocephala CIAT 17263; CayLd is a mixture of Cay2 and Leucaena diversifolia ILRI 15551. Hay is dried material of Dichantium aristatum*.

### Dry Matter and Nutrient Intake

The consumption of each plant fraction in the mixed diets was similar for both treatments. For the CayLl diet, animals consumed 77.5% grass and 22.5% legume, whereas, in CayLd, the animals consumed 81.8% grass and 18.2% legume. DMI was highest in diets that included the *Leucaena* forages (CayLl and CayLd), reaching values up to 2.94% of BW (*P* < 0.001), whereas the lowest DMI corresponded to the treatments of lower nutritional quality (Cay1 and Hay) (Table 2). The highest differences in nutrient intake were related to CP content. The treatments that included legumes provided an average of four times more CP than grass alone treatments (*P* < *0.001*).

**Table 2:** Average dry matter and nutrient intake in zebu steers fed with tropical forages of different composition and nutritional value.

	**Cay1**	**Cay2**	**CayLl**	**CayLd**	**Hay**	***P*-value**	**SEM**
DM, kg animal^−1^	1.93^c^	3.46^b^	5.77^a^	6.71^a^	2.36^c^	<0.0001	0.48
CP, kg day^−1^	0.10^d^	0.28^c^	0.56^b^	0.89^a^	0.14^d^	<0.0001	0.03
NDF, kg day^−1^	1.35^c^	2.36^b^	3.67^a^	3.82^a^	1.45^c^	<0.0001	0.31
ADF, kg day^−1^	0.76^b^	1.2^b^	2.07^a^	1.97^a^	0.91^b^	<0.0001	0.18
GE, Mj	31.87^d^	59.35^c^	96.96^b^	118.2^a^	33.6^d^	<0.0001	7.56

a,b,c,d, *mean values among the same row with different superscript are significantly different (P < 0.05); SEM, Standard error of the mean; BW, body weight; DM, dry matter; CP, crude protein; NDF, neutral detergent fiber; ADF, acid detergent fiber; GE, gross energy. Cay1 and Cay2 diets correspond to Urochloa hybrid cv. Cayman-CIAT BR02/1752 (Cayman) collected at a different time of regrowth (i.e., 65 and 45 days, respectively). CayLl is a mixture of Cay2 and Leucaena leucocephala CIAT 17263; CayLd is a mixture of Cay2 and Leucaena diversifolia ILRI 15551. Hay is the dried material of Dichantium aristatum*.

### Methane Emissions

**Figure 1:**
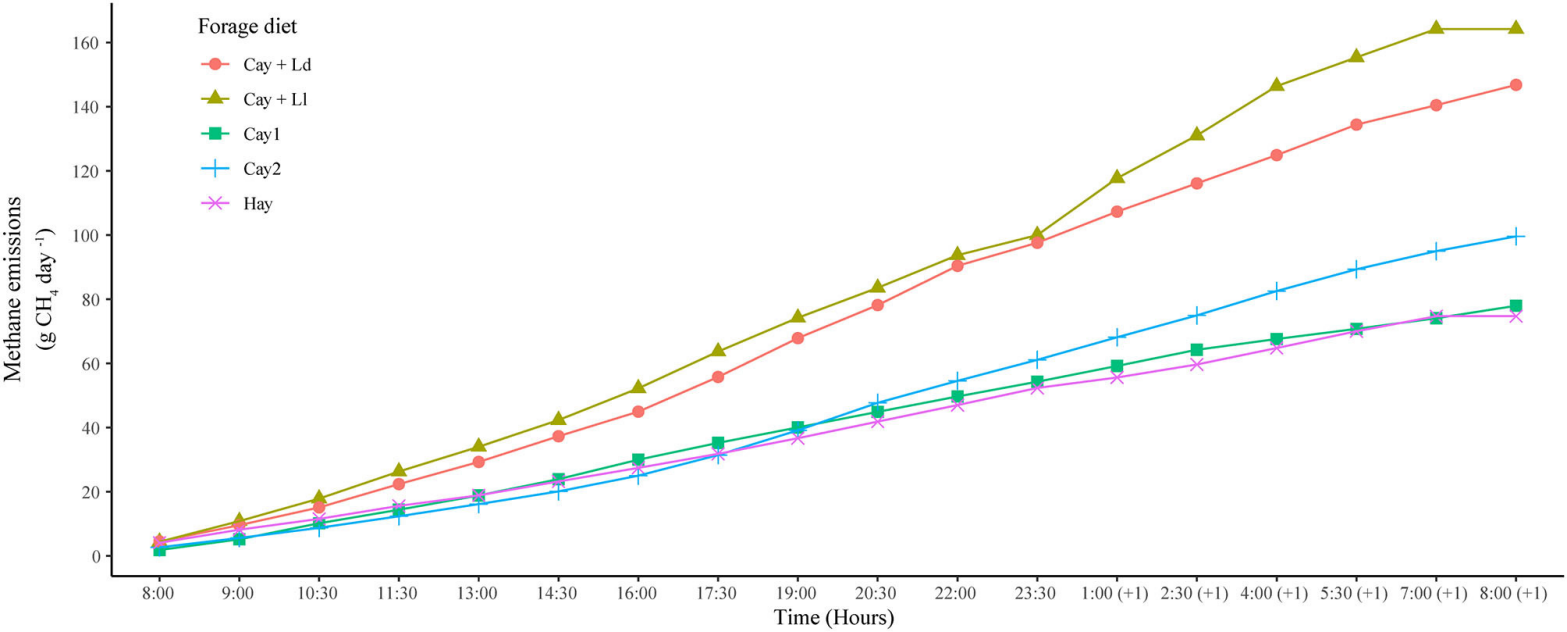
Accumulated methane emissions in zebu steers fed with tropical forages of different nutritional value.

The total CH_4_ accumulated during the 24-h measurement period ranged between 74.7 g and 164.2 g animal^−1^ and was statistically higher (*P* < *0.0001*) in treatments CayLl and CayLd that included the legumes (Figure 1). No significant differences were observed among the other grass alone treatments. Methane accumulated per hour varied between 4.4 and 10.2 g. When CH_4_ emissions were expressed per kg of DMI, kg of DMD, or organic matter digested (OMD), there was an opposite relationship to that obtained when CH_4_ emissions were expressed as g CH_4_ animal^−1^ day^−1^ (See Figure 1 and Table 3). The diets that had higher total emissions as g CH_4_ animal^−1^ day^−1^ (CayLl and CayLd) had the lowest emissions of CH_4_ per kg of DMI, kg DMD or OMD, and consequently, these were the treatments with the lower energy losses as a percent of GE intake (Ym).

**Table 3:** Daily production of methane in zebu steers fed with tropical forages of different composition and nutritional value.

	**Cay1**	**Cay2**	**CayLl**	**CayLd**	**Hay**	***P*-value**	**SEM**
CH_4_, g kg of DMI^−1^	60.39^a^	30.15^b^	27.57^b^	19.79^b^	35.98^b^	<0.0001	7.25
CH_4_, g kg OMI^−1^	68.52^a^	34.33^b^	31.46^b^	22.76^b^	45.16^a^	<0.0001	8.62
CH_4_, g kg DMD^−1^	118.38^a^	48.8^b^	45.17^b^	32.45^b^	75.17^a^	<0.0001	14.6
CH_4_, g kg OMD^−1^	133.98^a^	55.49^b^	52.71^b^	38.31^b^	94.25^a^	<0.0001	4.62
Ym, %	20.73^a^	9.76^b^	9.10^b^	6.22^b^	14.23^a^	<0.0001	2.66
Weight gain expected, g/day^*^	–(68)^d^	273^c^	497^b^	742^a^	141^c^	<0.0001	0.48
CH_4_, g/ g of LW gain	(0.85)^a^	0.36^c^	0.33^c^	0.20^c^	0.53^b^	<0.0001	0.32

a,b,c,d, *mean values among the same row with different superscript are significantly different (P < 0.05); SEM, Standard error of the mean; DMI, dry matter intake; OMI, organic matter intake; MW, metabolic weight; DMD, dry matter digested; OMD, organic matter digested; LW, live weight*.

* *Weight gain values calculated by CNCPS. Cay1 and Cay2 diets correspond to Urochloa hybrid cv. Cayman-CIAT BR02/1752 (Cayman) collected at different time of regrowth (i.e., 65 and 45 days, respectively). CayLl is a mixture of Cay2 and Leucaena leucocephala CIAT 17263; CayLd is a mixture of Cay2 and Leucaena diversifolia ILRI 15551. Hay is dried material of Dichantium aristatum*.

**Figure 2:**
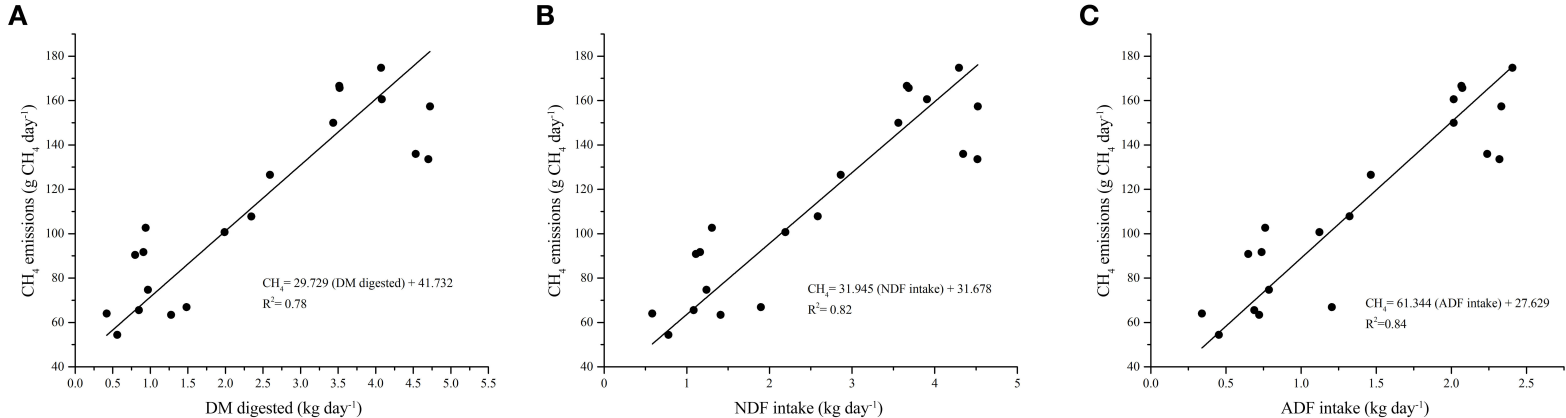
**(A)** Relationship between dry matter digested (kg animal day^−1^) and enteric methane emissions (g day^−1^). **(B)** Relationship between NDF intake (g kg DM^−1^) and enteric methane emissions (g day^−1^). **(C)** Relationship between ADF intake (g kg DM^−1^) and enteric methane emissions (g day^−1^).

**Figure 3:**
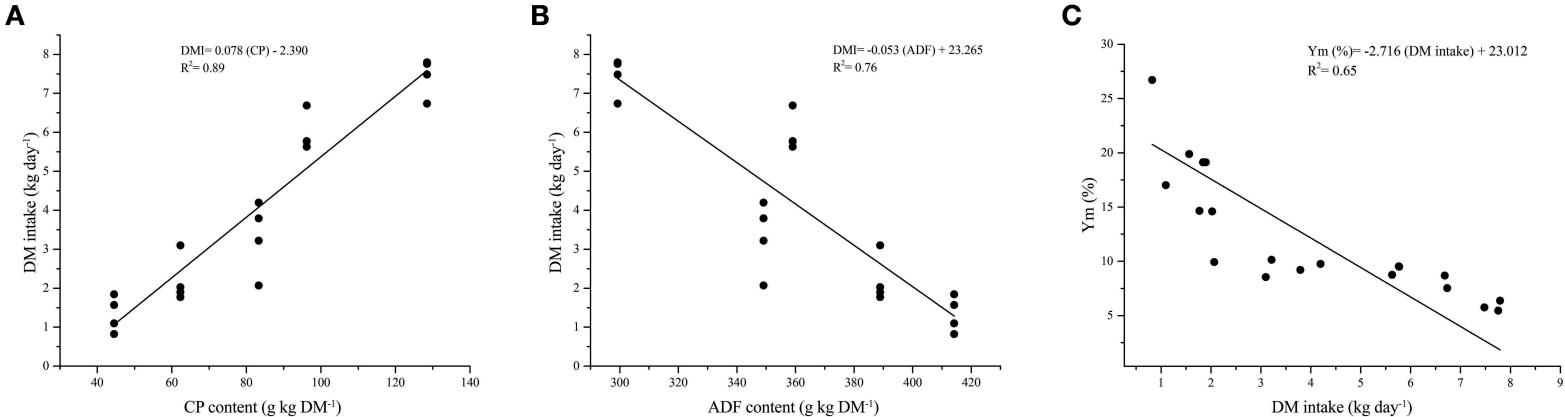
**(A)** Relationship between PC content (kg day^−1^) and dry matter intake (kg day^−1^). **(B)** Relationship between ADF content (kg day^−1^) and dry matter intake (kg day^−1^). **(C)** Relationship between Ym (%) and dry matter intake (kg day^−1^).

A strong positive relationship was observed between CH_4_ emissions and parameters like DMD (Figure 2A), NDF intake (Figure 2B), and ADF intake (Figure 2C). These relations resulted in calculations of CH_4_ emissions increasing 29.7 g CH_4_ animal^−1^ day^−1^ per each additional kg of DMD, 31.9 g CH_4_ animal^−1^ day^−1^ per each additional kg of NDF consumed, and 61.3 g CH_4_ animal^−1^ day^−1^ per each additional kg of ADF consumed. Furthermore, a strong positive relationship was found between DMI and the content of CP in the diet (Figure 3A); and a negative relationship was observed between DMI and fiber content in the five diets evaluated (Figure 3B). Precisely, the DMI increased by 78 g when the CP increased by 1 g kg^−1^ DM but decreased by 53 g for each kg of ADF contained in the diets evaluated.

During the experimental period, maximum internal temperatures recorded in the polytunnel ranged between 30–32.6°C and were reached at around 14:00 and 15:30 h. Minimum internal polytunnel temperatures ranged from 17–22.3°C and were recorded between 4:00–6:00 h. Whereas, the ambient temperatures were generally lower than those recorded in the polytunnel the differences did not exceed 1.5°C. Relative humidity (RH) values were always higher inside compared to outside the polytunnel. When the highest RH (~92%) were recorded inside the polytunnel, between 2:00 and 5:00 h, the outside RH values were ~84%.

### Expected Live Weight Gains

Expected live weight gains for animals in different treatments are shown in Table 3. Animals that consumed Cay1 were expected to lose weight, while for the other treatments, the weight gain ranged between 141 and 742 g day^−1^ for the Hay and CayLd diets, respectively. The amounts of CH_4_ emitted by animals that consumed Cay1 diet in relation to their live weight gain was calculated assuming that the animals transition from consuming this forage to consuming the Cay2 treatment forage which is of better nutritional quality. In this way the animals move from losing 68 g day^−1^ to gaining 205 g day^−1^.

## Discussion

### Nutritional Quality of Forage Based-Diets

Our results showed that an improvement in the nutritional quality in diets based on tropical forages was associated with an increase in DMI, especially when legumes were included in the diet. Diets based on feeds with high fiber contents (NDF and ADF) were less digestible, while those based on feed with low fiber contents were more digestible (Table 1). This observation is because the amount of fiber present in forages is directly related to the increase in resistance to the reduction of forage particle size and, therefore, inversely related to its digestibility (12). Likewise, the rate of passage of the feed is inversely related to its fiber content: a high fiber content decreases the potential for DMI by increasing the feed retention time in the rumen (37, 38).

The nutritional quality of the diet is a significant factor influencing DMI and productivity. Low nitrogen and high fiber contents may limit the DMI by the animal. These two variables showed a close relationship in the present study and showed that the high CP content (Figure 3A) and the lower fiber content (Figure 3B), resulted in an improvement of DMI by the animals. It is commonly accepted that in diets with 8–10% CP (grass-alone diets), consumption is limited by the capacity of the reticulum-rumen and the rate of passage of the intake, and if the diet exceeds 10% CP (*e.g.*, in grass-legume diets), consumption is probably affected by other metabolic factors (39).

### Nutrient Intake and Digestibility

Dry matter intake is regulated by digestive and metabolic factors, such as the reticulum-rumen fill, osmolality, and kinetics, as well as specific digestive hormones and blood metabolites derived from feed digestion or catabolism (Forbes, 2000). Rumen filling is related to the fiber concentration of the feed consumed by the animal, which could explain our results regarding the decrease of DMI with the increase of the fiber concentration (Figure 3B). However, the filling effect of diet also depends on factors affecting digestion rates and fluxes of the reticulum-rumen, such as particle size and density and intake of NDF (40). Since NDF intake is directly related with CH_4_ emissions (41, 42), the intake of fiber may explain the CH_4_ production observed in treatments with higher NDF in this current study (Figure 2B).

It is important to highlight that the leaves of forages such as *Leucaena* have been reported to contain mimosine (2.3–12% DM) which, depending on their concentration, can modify ruminal fermentation and, thus, CH_4_ emissions (43, 44). An *in-vitro* study conducted in Brazil by Soltan et al. (45) reported that mimosine stimulated acetogenesis as an alternative to methanogenesis in the rumen, leading to a reduction in CH_4_ emissions without adverse effects on nutrient degradability. The cited study was however inconclusive on the role of mimosine in CH_4_ reductions (45). In the present study, the concentration of mimosine in *Leucaena* forage was not determined. Therefore, future research need to explore the effects of mimosine on CH_4_ production, ruminal fermentation parameters, nutrient consumption, and degradability.

The CT contents of the two *Leucaena* species did not appear to have adverse effects either on digestibility or DMI of the evaluated diets. The CT content of the two diets that included *Leucaena* did not exceed 50 g kg DM^−1^, which has been reported as the threshold CT content beyond which palatability is reduced (46). Our findings corroborate with Montoya-Flores et al. (47) that reported that the inclusion of 12% *L. leucocephala* leaves in a grass-based diet resulted in a CT contents of 0.27% which was enough to increase the digestibility of dietary protein and organic matter while reducing CH_4_ production, without adverse effects on the microbial populations and rumen fermentation. On the other hand, Piñeiro-Vasquez et al. (48) reported that *L. leucocephala* as a source of CT has an optimal inclusion level of 20–40% of the DM on the ration and in this range can reduce CH_4_ emissions and improve nitrogen intake without affecting DMI.

In the current study, offering diets with low protein content, high fiber and low digestibility led to depressions in DMI and to lower CH_4_ emissions per animal per day (Figures 2, 3), but higher CH_4_ emissions when expressed per unit DMI, DMD and OMD (Table 3). Forage intake and digestibility are some of the most important factors limiting the production of cattle grazing in tropical forages, and it has been reported that when the diets have <6% of CP, there are restrictions to cattle intake and productivity due to a protein deficiency (49, 50). Hence, it is critical to identify strategies to increase both nutrient intake and proper forage grazing. From the results of this investigation, it can be concluded that the dietary inclusion of both *L. leucocephala* and *L. diversifolia* is a suitable option to increase cattle productivity under tropical conditions.

The voluntary intake of forage and nutrients was higher for the Cayman*-Leucaena* diets (CayLl and CayLd), being up to 2.97% of BW (Table 2). Similar results were reported by Molina et al. (17), who observed DMI of 2.47% of BW when *Leucaena* was included in the diet compared to 2.02% in a grass only diet. Likewise, Cuartas et al. (16) reported a voluntary DMI of 2.60% of BW when animals were offered intensive silvopastoral systems diets consisting of 31% *L. leucocephala* and 69% *Megathyrsus maximus*. For an intensive silvopastoral system with *L. leucocephala*, Gaviria-Uribe et al. (15) reported intakes of 2.46% of BW of a diet with 53.5% digestibility. These results suggest that the inclusion of legumes can be a strategy to increase DM and nutrient intake in cattle and, consequently, productivity, ameliorating a fundamental limitation of tropical cattle production, the consumption of low-quality diets that hinder a transition toward a sustainable intensification process.

There are several reports on the nutritional and productivity benefits of including *L. leucocephala* in the diet of grazing beef and dairy cattle (51–55). However, the current study is the first to report the benefits of including *L. diversifolia* in the diet of grazing cattle in Latin America. The two legumes used in the current study enabled us to evaluate feed options adapted to different soil conditions, altitudes and similar precipitation. In its native range, *L. leucocephala* grows in alkaline soils (pH 7.0–8.5) and altitudes up to 2,000 masl, whereas *L. diversifolia* grows in mildly acid soils (pH 5.5–6.5) below 1,500 masl. Only *L. leucocephala* tolerates long dry seasons (of up to 7 months). On the other hand, *L. diversifolia* is well-adapted to low temperatures at which *L. leucocephala* ceases to grow (16°C) (56). In terms of nutritional quality*, L. diversifolia* can have CP values of 20–25% with a digestibility of 60%, while *L. leucocephala* has CP values of between 12 and 25% with a higher digestibility (65–85%) (57).

### Methane Emissions

It has been reported that cattle with higher consumption of organic matter are associated with a greater physiological capacity of the rumen, longer retention times, and higher digestion rates, increasing CH_4_ production (58, 59). Similar findings were observed in the current study, where the amount of CH_4_ that was emitted was related to the DMD (Figure 2A), NDF (Figure 2B), and ADF intake (Figure 2C). High NDF and ADF intake increased CH_4_ emissions and conversely, greater degradability of DM and OM resulted in lower CH_4_ emissions. The DMI and the composition of the diet have a major impact on enteric CH_4_ production, and many of the CH_4_ emission reduction strategies are focused on handling these two components (60, 61), which is aligned with our observations.

The variables of DMI, nutrient content, and CH_4_ emissions were strongly related to each other (Figures 2, 3). Treatments with higher nutritional quality, *i.e.*, higher digestibility, higher CP content, and lower FDN and FDA content had higher DMI and lower CH_4_ emissions and, therefore, lower energy loss in the form of CH_4_. As a consequence, the intensity of these emissions was lower when related to animal production parameters (Table 3).

It is relevant to note that although Cay1 and Cay2 diets corresponded to the same grass, large differences were observed in their nutritional quality due to different harvesting times (Table 1) that aimed at stimulating two grazing management practices. These differences directly impacted the voluntary DMI (Table 2) and CH_4_ emissions from each of these treatments (Table 3). As expected, Cay2 had a higher nutritional quality, DMI, and resulted in lower CH_4_ emissions compared to Cay1, most probably due to higher lignification in Cay1. When Cay2 was compared to treatments associated with *Leucaena* (CayLl and CayLd), no significant differences in CH_4_ emissions (g of CH_4_ per kg of DM) were observed (Table 3). Furthermore, forage yield and their nutritional quality are influenced by numerous factors such as biotic and abiotic conditions and management practices, namely cutting (including grazing and browsing) frequency, plant maturity, climatic conditions, and soil fertility, among others. A longer maturation time of the grass results in lower protein content and higher fiber and lignin contents (9).

In this sense, proper grazing management of a pasture determines its nutritional quality and, therefore, the productivity parameters of the animals, including CH_4_ generation. Improving the quality of the fodder consumed by the animals is a feasible option to mitigate CH_4_ emissions, in which absolute emissions may increase, but improved animal performance decreases the intensity of emissions. However, grazing management needs constant adaptation as it is highly dependent upon weather and other environmental factors, and their adoption is limited by technical knowledge and its transfer (60, 62).

The Hay treatment was included as natural pastures are highly abundant in livestock systems in the tropics. Hay from natural pasture is one popular option for conserving forage for times of limited forage availability (*e.g.*, extended drought periods). The high values of CH_4_ emissions in this treatment, low digestibility, and consumption by the animals were mainly related to its nutritional quality (Tables 1, 3). In general, naturalized pastures produce more CH_4_ emissions as influenced by DMI, degradation, and passage rates (63). However, an important factor in hay preparation and storage is to keep the material dry. If humidity is present, problems of plant material deterioration can occur, which could also affect the fodder quality and consumption by animals.

Treatments that had higher DMI had the highest CH_4_ emissions (g day^−1^) (Figure 1). However, when analyzing these emissions per unit of DMI and DMD, this relationship changed, and the treatments with higher DMI (CaLl and CaLd) showed lower emissions per unit of DMI and DMD (Table 3, Figure 2). Similar findings have been reported by various authors (10, 64). In addition, Montenegro et al. (65) also observed reductions in CH_4_ emissions of 25% (31 *vs*. 23 g CH_4_ kg DMI^−1^) when the animals consumed more DM of high-quality diets. It has been reported that CH_4_ emissions can increase as a result of increased DMI, but the intensity of emissions per unit product would also be reduced (17, 58, 60, 66) probably as a result of the better nutritional quality and digestibility of these diets (58, 67–69). These observations show the importance of adequately managing grazing and browsing regimes of forages as this could influence not only DMI but also CH_4_ emissions. In turn, improved grazing management techniques are a mitigation strategy with potential on open grazing cattle systems.

Both tree legume species presented similar CT, DMI, and DMD. However, *L. diversifolia* seems to have a greater potential for reducing CH_4_ emissions than *L. leucocephala* (Table 3). Several previous studies have reported that the factors influencing the concentrations of tannins in plants include plant genotype, tissue developmental stage, environmental conditions, plant part, soil fertility and the method of forage collection (70–73). Likewise, the effect on CH_4_ emissions and the nutritional effects of CT are also highly influenced by their structural characteristics, including the composition of proanthocyanidins and molecular weight (74).

Analysis of the energy losses in the form of CH_4_ observed in this study suggests that in the lower quality diets, this loss is much higher due to the maintenance requirements of metabolizable energy of animals, and this proportion decreases with better quality diets (Table 3). Hence, with increased diet quality, more energy will be available for productive processes, effectively reducing the intensity of CH_4_ emissions. The “Methane conversion factor,” also known as “methane yield” (Y_m_), considers the CH_4_ emitted per unit of feed energy intake when both variables are expressed as the energy of combustion (75). When fed with grass of better nutritional quality (Cay2), animals had improved efficiency in the utilization of feed and, therefore, lower energy loss in the form of CH_4_, as suggested by Blaxter and Clapperton (76).

Methane yield values obtained for most treatments exceed the value suggested by the (77) (7.2%) (78), but some are similar to those reported by other authors for cattle fed in tropical countries. For example, (67) reported Y_m_ values ranging between 6.7% and 11.4% in mature *Brahman* cattle fed with diets based on tropical forages and (17) reported Y_m_ values of 7.96 in cattle consuming a 74% *Cynodon plectostachyus* and 26% *L. leucocephala* diet, and of 9.42 when receiving a 100% *C. plectostachyus* diet. Also, Molina-Botero et al. (79) reported that heifers supplemented with *Gliricidia sepium* foliage mixed with ground pods of *Enterolobium cyclocarpum* presented less GE loss in the form of CH_4_ (Y_m_ = 7.59%) than those fed with grass-alone (Y_m_ above 8.1%). In those reports and the present study, it seems improving the quality of the diets offered, resulted in more efficient use of consumed energy, which results in lower Y_m_ values. Increasing forage intake improves feed conversion efficiency because CH_4_ losses, as a proportion of the energy consumed are reduced (50, 67). The energy use efficiency of diets can also be observed from the relationship between Y_m_ and DMI (kg d^−1^). In this study, superior energy losses as a percentage of GE in the form of CH_4_ occurred when DMI decreased (Table 3).

Other authors have reported similar results to those observed in this study, in which the proportion of Y_m_ increases in diets high in fiber (80–82). It is essential to consider that in this study, there was very low DMI as a result of offering a very low-quality grass in Cay1 (low-quality *Urochloa* hybrid cv. Cayman). The very low-quality grasses are generally not typically used for experimentation. For this reason, the exceptionally high Y_m_ values obtained with the low-quality Cayman grass, although not unexpected, are difficult to compare to other reports.

### Expected Live Weight Gains

According to the expected live weight gains with the different treatments, animals with such a low level of DMI and nutritional value observed in Cay1 are expected to lose weight. However, in a real situation, the animals would not lose weight for the rest of their productive stage. In general, cattle fed with tropical pastures present a lower live weight gain than cattle fed with temperate pastures. Nevertheless, growth stages are different in the animal's life, and live weight could be more evident in some of them (83).

There was a clear significant difference in the expected live weight gain and a significant difference in CH_4_ emissions between treatments with *L. leucocephala* and *L. diversifolia*. Treatments like Cay2 and CayLl did not significantly differ in the amount of CH_4_ emitted (g g of live weight gain^−1^), however, expected weight gain was higher in CayLl, so the emissions intensity was lower for this treatment. A study conducted in Australia reported that the introduction of legumes generally resulted in an annual live weight increase of 60 kg (83). Also, *Leucaena* trees which seem to improve nutritional value and add nitrogen to the soil, improve grazing resistance and longevity (84), can be used in intensive rotational grazing systems (52) and are reported to be the most cost-effective option for a finishing period at the end of the growth path (85).

### Perspectives

Colombia has the ambition of reducing the economy-wide GHG emissions by 20% (86). As a major GHG emitter, the cattle sector is called to cut emissions in order to achieve these targets. As suggested by our findings, there is an important variation in CH_4_ emissions associated with cattle feed options. To account for this variation in national GHG inventories, there is a need to move to higher Tier emission estimation methods (Tier 2 to Tier 3) with the IPCC guidelines. Results from the current study indicate that grazing management that accounts for the age of the pasture could be a viable GHG mitigation option. However, while in our study the inclusion of *Leucaena* sp. did not result in significant reductions of total CH_4_ emissions, farmers could benefit from higher live weight gains compared to grass alone pastures. This efficiency improvement indicates a reduction in CH_4_ emission intensities (kg of CH_4_ per kg of meat). Additionally, and based on our experimental data, farmers can benefit from superior live weight gain rates by adopting *L. diversifolia* over *L. leucocephala*.

## Conclusions

The nutritional quality of the diet offered to the cattle directly influences the voluntary intake and CH_4_ emissions generated. In the present study, the diets with the highest dry matter intake were those where *Leucaena* was included. Likewise, cattle fed with grass harvested at 45 days of regrowth resulted in a lower energy loss as methane and higher dry matter intake compared to cattle fed with grass harvested at 65 days of regrowth. Also, although CH_4_ emissions from an adequately managed pasture were similar to those emitted from diets that include *Leucaena*, the legume-based systems offer additional advantages in DM that are reflected in the higher live weight gains of cattle, so the intensity of the CH_4_ emissions generated in legume-based system were lower and making these systems a good option to implement for transitioning toward sustainable tropical cattle production.

## Data Availability Statement

The raw data supporting the conclusions of this article will be made available by the authors, without undue reservation.

## Ethics Statement

The animal study was reviewed and approved by Ethics Committee of the International Center for Tropical Agriculture (CIAT) and following protocols of the Colombian law No. 84/1989.

## Author Contributions

XG-U, DB, TR, NC, RB, and JA: conceptualization and methodology. XG-U, DB, and RB: formal analysis. XG-U: writing – original draft preparation. XG-U, DB, TR, IM-B, NC, RB, and JA: writing – review & editing. DB, RB, and JA: supervision. JA: project administration. NC and JA: funding acquisition. All authors contributed to the article and approved the submitted version.

## Conflict of Interest

The authors declare that the research was conducted in the absence of any commercial or financial relationships that could be construed as a potential conflict of interest.
